# The Human Immune Response to Cadaveric and Living Donor Liver Allografts

**DOI:** 10.3389/fimmu.2020.01227

**Published:** 2020-06-22

**Authors:** Angus Hann, Daniel-Clement Osei-Bordom, Desley A. H. Neil, Vincenzo Ronca, Suz Warner, M. Thamara P. R. Perera

**Affiliations:** ^1^The Liver Unit, Queen Elizabeth Hospital Birmingham, Birmingham, United Kingdom; ^2^Institute of Immunology and Immunotherapy, University of Birmingham, Birmingham, United Kingdom; ^3^Department of Cellular Pathology, University Hospitals Birmingham NHS Foundation Trust, Birmingham, United Kingdom; ^4^The Liver Unit, Birmingham Children's Hospital, Birmingham, United Kingdom

**Keywords:** liver, transplant, immunity, tolerance, rejection, cadaveric, living donor

## Abstract

The liver is an important contributor to the human immune system and it plays a pivotal role in the creation of both immunoreactive and tolerogenic conditions. Liver transplantation provides the best chance of survival for both children and adults with liver failure or cancer. With current demand exceeding the number of transplantable livers from donors following brain death, improved knowledge, technical advances and the desire to prevent avoidable deaths has led to the transplantation of organs from living, ABO incompatible (ABOi), cardiac death donors and machine based organ preservation with acceptable results. The liver graft is the most well-tolerated, from an immunological perspective, of all solid organ transplants. Evidence suggests successful cessation of immunosuppression is possible in ~20–40% of liver transplant recipients without immune mediated graft injury, a state known as “operational tolerance.” An immunosuppression free future following liver transplantation is an ambitious but perhaps not unachievable goal. The initial immune response following transplantation is a sterile inflammatory process mediated by the innate system and the mechanisms relate to the preservation-reperfusion process. The severity of this injury is influenced by graft factors and can have significant consequences. There are minimal experimental studies that delineate the differences in the adaptive immune response to the various forms of liver allograft. Apart from ABOi transplants, antibody mediated hyperacute rejection is rare following liver transplant. T-cell mediated rejection is common following liver transplantation and its incidence does not differ between living or deceased donor grafts. Transplantation in the first year of life results in a higher rate of operational tolerance, possibly due to a bias toward Th2 cytokines (IL4, IL10) during this period. This review further describes the current understanding of the immunological response toward liver allografts and highlight the areas of this topic yet to be fully understood.

## Introduction

At present, orthotopic liver transplantation (OLT) is the only effective treatment option for many conditions (1). Unfortunately the demand for organs exceeds the supply, each year in the United Kingdom ~15% of patients awaiting a liver transplant either die or are delisted due to disease progression (2). Improvements in surgical technique, graft preservation techniques, perioperative care and immunosuppression has resulted in better short term graft function and patient survival (1). The detrimental effects of long term immunosuppression in regards to malignancies, metabolic disturbances, cardiovascular disease, renal failure and opportunistic infections are well-recognized (3, 4). These contribute significantly to the longer term morbidity and mortality in transplant patients (5, 6). The withdrawal of immunosuppression would eliminate these complications and is therefore highly desirable (7). The term “operational tolerance” implies a state of stable graft function following cessation of immunosuppressive medications and without evidence of rejection or graft injury (6). Operational tolerance is known to occur spontaneously following OLT more frequently than any other solid organ transplant (8). Immunosuppression withdrawal trials suggest that the rate of spontaneous operational tolerance may be as high as 40% in adults and 60% in pediatric patients post OLT (4, 9). Research focused on detecting biomarkers that identify patients who have a higher probability of developing operational tolerance are ongoing, as this would allow an expedited withdrawal of immunosuppression (10). However, a major aim in the field of transplantation is the development of tolerance inducing therapies. Therapeutic administration of interleukin-2 (IL-2), Regulatory T cells (Tregs), and dendritic cells (DC) are all being investigated, some of which are in phase II clinical trials. Further advancement in this area requires a detailed understanding of the immunophysiology of the liver and the interaction with the systemic immune system.

The allograft implanted during OLT can be from either a living or deceased donor. Procurement of a deceased individuals organs can occur following brain death (DBD) or cardiac death (DCD) and the organ can be split between two recipients depending on the volume of parenchyma required. Deceased donors are scarce in many countries and implanting organs from different ABO blood groups may be the only option to save a recipient's life, this is known as ABO incompatible (ABOi) liver transplantation. Early reports of ABOi OLT utilizing conventional immunosuppressive regimes and deceased donors yielded significantly inferior results (11). However, the introduction of modern therapies such as rituximab, a chimeric monoclonal antibody against the protein CD20 on B lymphocytes, has enabled living donor ABOi OLT to be common practice in many countries with equivalent results to conventional living donation (11). Liver transplantation for pediatric patients is more challenging due to lack of size matched donors. Pediatric patients most commonly receive a segmental graft that could be from a split, reduced size or living donor liver transplant (12). It is likely that variations in both the graft types and indications for OLT influence the immune response elicited. Understanding these in detail will allow further refinement of immunosuppressive regimes and tolerance inducing therapies.

## Methods

Relevant existing publications for this narrative review were identified by searching the Pubmed, EMBASE and Medline databases. The search was limited to the English language, but no other filters were utilized. The following terms were utilized (in a variety of combinations); *liver, transplant, immune response, innate, acquired, cell, antibody, rejection, ischaemia reperfusion, cadaveric, living donor, ABO incompatible, pediatric, pediatric*. Any additional publications relevant to this review were then identified by manually searching article reference lists.

## Immune Function of the Liver

The liver is one of the two organs in human body with a dual blood supply, deriving blood from both arterial and portal venous blood. Therefore, it is exposed to both systemic and enterically derived pathogens (13). Portal venous blood delivers essential gut derived nutrients to the liver, however it also contains a significant volume of foreign antigens (13). Once a pathogen breaches our first defensive barrier, the intestinal epithelium, it will travel to the liver and therefore this organ is essential in the defense against harmful pathogens (14). However, unrestrained immune activation against non-pathogenic foreign antigens would have a detrimental result. The liver has a unique “tolerogenic” property which prevents this occurring. A large population of immune cells reside in the liver including macrophages (Kupffer cells), lymphocytes and dendritic cells. In addition, both the hepatic stellate cells (HSCs) and hepatocytes have immune functionality. Under certain inflammatory conditions, hepatocytes can express MHC II molecules and along with HSCs have been shown to interact with lymphocytes (13). The immune surveillance and pathogen clearance within the liver occurs predominantly at the hepatic sinusoids (14).

Systemic infection has a significant effect on the liver. Sepsis is known to induce changes in gene and protein expression and this alteration in hepatocellular function is known as the acute phase response. This response is triggered by IL-6 and IL-1 from monocytes and stimulates hepatocytes to release numerous acute phase proteins (APPs) (14). A number of these proteins then proceed to augment the systemic immune system by opsonising, further cellular activation or via direct action of complement (14). APPs have a further role in the abrogation of the immune response to prevent tissue injury from an over response (15). It has been shown that APPs such as serum amyloid A and Cxc11/KC result in the mobilization of myeloid derived suppressor cells (MDSCs) which suppress inflammation and T cell responses in particular (15). Bacteraemia is reported to be ten times more common in patients with cirrhosis and it is associated with a fourfold increase in mortality in comparison to those without cirrhosis (14, 16). An imbalance of both the defense mechanisms and counterregulatory responses are likely contributory to the susceptibility of these patients to life threatening sepsis.

The liver is also a target of multiple autoimmune diseases. Autoimmune liver disease (AILD) is comprised of autoimmune hepatitis (AIH), primary biliary cirrhosis (PBC), and primary sclerosing cholangitis (PSC). AIH results from a T cell mediated insult on autoantigens and causes a chronic hepatitis with an interface and lobular hepatitic component, however the portal/interface component usually predominate (17). A subset of T-cells, known as Tregs (CD4^pos^, CD25^high^, CD127^LOW^, FoxP3^pos^), are key components of the immunosuppressive arm of the immune system, suppressing effector cell activity and restoring immune homeostasis. Multiple immunosuppressive mechanisms have been attributed to Tregs such as the secretion of anti-inflammatory cytokines and inhibitor molecules (e.g., CTLA4), depletion of crucial growth factors, disruption of effector cell metabolism by promoting the accumulation of adenosine nucleosides, consuming scarce amino acids and also by direct cytotoxic killing of effector cells (18). The number and function of Tregs are reduced in AIH, giving rise to the theory of unchecked or un-inhibited effector cell activity perpetuating the inflammatory cascade. AILD and liver allograft rejection both rely on leukocyte recruitment to the liver, and subsequent migration from the vasculature into the tissue. In most tissues, migration across the vascular endothelium occurs at post capillary venules (19). However, a study utilizing intravital microscopy demonstrated that in 80% of leukocytes adhere to the endothelium in the hepatic sinusoids and this is where the majority of leukocyte extravasation occurs in the liver (19, 20). Shear stress in the sinusoids in low and therefore the “rolling” process described for leukocyte extravasation is not required (19). Recruitment and adhesion of leukocytes is enhanced by hepatic sinusoidal endothelium expressing peptide molecules vascular adhesion protein 1 (VAP-1), VCAM-1, ICAM-1, CD44 (19). The recruitment of lymphocytes (in particular Th2) to the liver is enhanced by VAP-1. An additional molecule known as the common lymphatic endothelial and vascular endothelial receptor 1 has been demonstrated to recruit Tregs to the liver and promote transendothelial migration (19, 21). These recruitment mechanisms used by the liver are preserved after transplantation (19). The grafts endothelial cells are the first donor cells to encounter recipient leukocytes and their activation is likely an early event that leads to immune cell migration into the graft (22).

A hepatic allograft has immunoprotective benefits. The frequency of renal allograft TCMR is significantly lower in combined liver-kidney recipients in comparison to kidney alone recipients (23). In addition, less frequent and severe episodes of renal allograft rejection have been demonstrated when kidney transplants occurred in patients with previous liver transplants (24) Similar immunoprotective benefits were less pronounced when renal transplants followed heart and lung transplantation (24). Suggested mechanisms of protection are immune exhaustion due to high antigen burden, chimerism, and T cell deletion within the liver (23, 24). Chimerism refers to the presence of donor cells within the recipient's circulation and occurs due to cell migration from the graft (23, 25). Hematopoietic and T cells from the liver allograft migrate into the recipients circulation and if donor cells comprise more than 1% of the tissue it is referred to as macrochimerism, if they comprise <1% it is known as microchimerism (23). The persistence of chimerism has been associated with less rejection and is postulated to have a role in tolerance induction (25). T-cell deletion is suggested to occur within the liver due to direct contact with parenchymal cells (23). It has been suggested by Abrol et al. that the tolerance inducing effect of the liver in combined liver-kidney transplantation is due to a cell type from within the donor liver migrating to the other transplanted organ and inducing immune regulatory effects (23).

## The Different Types of Liver Transplant Allografts

**Figure 1:**
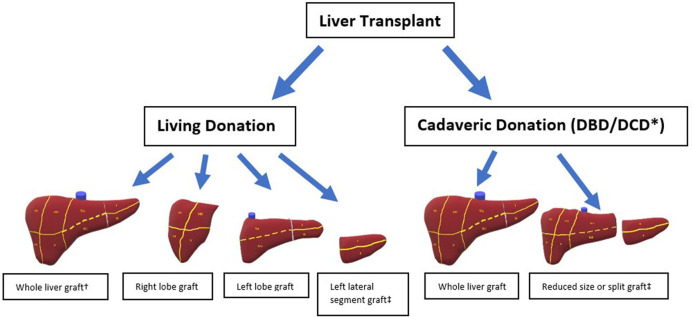
Different types of liver allografts. ^*^ DCD grafts not split. ^†^ Living donation of whole liver only possible with domino transplantation. ^‡^ Either of these grafts is suitable for an auxiliary transplant.

The first liver transplant recipient to survive more than 24 h following the procedure was a 19-month-old infant who received a whole liver graft obtained from a 18-month-old brain dead donor (26). This pivotal procedure was performed by Thomas Starzl and his team in 1967 and subsequently numerous different types of liver grafts have been utilized by transplant clinicians. The main initial distinction between grafts is whether they were obtained from a deceased or living donor (Figure 1).

A liver obtained from a deceased individual is known as a deceased donor graft and depending on the terminal event, these donors can be considered to have experienced brain death (DBD) or circulatory death (DCD). The universal definition of brain death is “The irreversible loss of the capacity for consciousness, combined with irreversible loss of the capacity to breathe and therefore irreversible cessation of the integrative function of the brainstem” and strict neurological criteria need to be satisfied to make this diagnosis (27). DBD donors therefore have spontaneous cardiovascular activity providing organ perfusion and are receiving mechanical ventilation, both of which are maintained until cold perfusate is administered to the graft. DCD donors are individuals who do not meet the strict brain death criteria but are receiving life sustaining treatment that is deemed to provide no overall benefit. In this scenario the organ support is withdrawn, and death is determined by the standard cardiorespiratory criteria. Therefore, DCD donation involves a period of circulatory arrest with resultant ischaemia prior to cold perfusion of the graft. This is known as the donor warm ischaemic time (dWIT). The modified Maastricht criteria (Table 1) is used to sub classify the DCD donors based on the time of expected dWIT, and safety of organs used in transplantation (28). Deceased donor grafts can be implanted as whole or split grafts. Splitting of a cadaveric graft in most instances would provide an extended right lobe graft to an adult and a left lateral section to a pediatric recipient. Due to supply not meeting demand, the use of grafts from cadaveric donors with suboptimal features are known as extended criteria donors (ECD) (29). These include advanced donor age, graft steatosis and a DCD donor which are all features associated with poorer transplant outcomes (29).

**Table 1:** Modified Maastricht criteria for donation following cardiac death.

	**Sub-category**	**Description**
Category I—Found dead (Uncontrolled)	IA	Unexpected cardiac arrest out of hospital without attempted resuscitation
	IB	Unexpected cardiac arrest in hospital without attempted resuscitation
Category II—Witnessed cardiac arrest (Uncontrolled)	IIA	Unexpected cardiac arrest out of hospital with unsuccessful resuscitation
	IIB	Unexpected cardiac arrest in hospital with unsuccessful resuscitation
Category III—Withdrawal of life support (Controlled)		Expected, planned cardiac arrest after withdrawal of care
Category IV—Cardiac arrest whilst brain dead (Uncontrolled, controlled)		Sudden cardiac arrest following brain death but prior to planned organ recovery

*Categories used to classify donation following cardiac death (22)*.

Living donor liver transplantation (LDLT) occurs when a live individual undergoes a partial hepatectomy and donates this portion of their liver. The type of hepatectomy will depend on the volume of parenchyma the recipient requires. The donor can be either biologically related or biologically unrelated. All living donor grafts will be partial grafts, the only exception to this would be when “Domino” transplantation occurs. A “Domino” transplant occurs when recipient A undergoes a total hepatectomy and this explanted liver is transplanted into recipient B, recipient A can survive as they receive a separate cadaveric or living donor graft (30). This strategy is possible when recipient A suffers from one of several hereditary metabolic diseases as these livers are otherwise normal. Familial Amyloid Polyneuropathy is the most common reason for domino liver transplantation (30). An auxiliary liver transplantation is another type of graft in which either a remnant or the entire native liver is left within the recipient (31). Auxillary transplantation is most commonly used in the setting of acute liver failure as a “therapeutic bridge” until the native liver regenerates (31).

## Post Reperfusion Syndrome and Preservation-Reperfusion Injury

**Figure 2:**
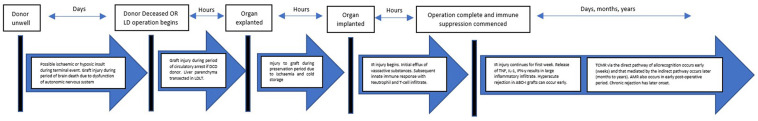
Pathway of a graft from donor to recipient. The journey of a liver allograft from donor to recipient. LDLT, Living donor liver transplantationl; IR: Ischaemia reperfusion; TCMR, T-cell mediated rejection, AMR, Antibody mediated rejection.

An intense inflammatory response occurs immediately post OLT due to multiple factors including surgical stress, tissue trauma, preservation-reperfusion injury (PRI), blood loss and alloantigen recognition. Traditionally the liver grafts are preserved *ex-situ* in cold storage, thus without perfusion or oxygen delivery. These preservation conditions minimize oxidative phosphorylation and reduce metabolic activity to ~10% of the normal rate, the energy of which is mainly derived by anaerobic metabolism (32). In addition to ischaemia, hypothermic preservation conditions have a deleterious effects on the cell organelles, cytoskeletons and membranes (33). Re-establishment of blood flow results in the release of reactive oxygen species (ROS) from the mitochondria which in turn cause the release of proinflammatory cytokines from Kupffer cells (34, 35). This predominantly innate immune response is known as PRI and is also characterized by liver sinusoidal endothelial cell (LSEC) dysfunction (35). Intraoperative cardiovascular instability can occur immediately following re-establishment of blood flow due to a large efflux of metabolic substrates from the damaged liver, this entity is known as postreperfusion syndrome (PRS) (36). Release of cytokines (Tumor necrosis factor-α, IL-1, Interferon-γ, tumor necrosis factor-β) results in the accumulation of neutrophils (35). Previous literature has suggested that the immunogenicity of the graft is increased with PRI due to interactions between the innate and adaptive immune system (37). Enhanced T-cell priming is thought to result from this interaction and contribute to both acute and chronic rejection (37). Advanced donor age, graft steatosis and prolonged cold ischaemic time are associated with more severe PRI manifestations (38). PRI has physiological consequences and is considered the main cause of primary non function (PNF) and delayed graft function (DGF) (34, 39). In livers with severe PRI, ~40% will manifest PNF (40). Figure 2 further demonstrates how the different events in the transplant process relate to the immune response.

The human immune system is commonly divided into innate and adaptive components with separate effector cells and activation pathways. However, evidence suggests third division of the immune system referred to as “innate-like” exists and is comprised of both B and T lymphocyte subsets (41). A characteristic of these cells is a rapid and robust response to antigens with limited memory capabilities (41). Natural Killer T cells (NKT) are one type of innate-like cell that is present in the liver sinusoids and has been implicated in the transplant PRI process (42). NKT cells are subclassified into type I and type II based on the expression of invariant TCR-α and minimal TCR-β (Type 1) in comparison to diverse TCR-α and TCR-β (type II) (42). In a murine experimental model of PRI, type I NKT cells were found to induce injury and with an increased intracellular expression and secretion of IFN-γ. Type II NKT were shown to be protective against PRI and the proposed mechanism was that they inhibit the pro-inflammatory effects of type I NKT cells (43).

## Liver Allograft Rejection

Acute T-cell mediated rejection (TCMR) is the most common immune mediated complication following liver transplantation (44). Less frequent immune complications are recurrence of an AILD, plasma cell rich rejection, antibody mediated rejection (AMR) and unresolved TCMR/AMR progressing to chronic rejection. Allorecognition of transplanted tissue is known to occur via three pathways; direct, indirect and semi-direct (45). The direct pathway involves the recipients T-cells recognizing the donor MHC molecules on donor antigen presenting cells (APCs). The indirect pathway occurs when the donor antigen is processed by recipient APCs and recipient MHC molecules expressed. The semi direct pathway involves cell exchange either via exosomes or the process of trogocytosis, which is the active transfer of plasma membrane fragments from an antigen presenting cell to a lymphocyte via cell conjugation (45, 46). The semi-direct pathway is yet to be completely understood but it is believed to involve the transfer of complete MHC-peptide complexes from donor APCs to recipient APCs. This results in a recipient APC displaying both a self and donor MHC molecule, both with an attached donor antigen. This brings both the direct and indirect pathway together onto a single APC and allows additional interaction between the two CD-4 or CD-8 T-cells that bind with each MHC:peptide complex, therefore forming a 3 cell model (45). All pathways lead to increased secretion of IL-2 and other inflammatory cytokines which induce T-cell proliferation. The initial alloreactive T cell response is driven by the direct pathway with the indirect pathway assuming the main role as time progresses (8, 45).

The diagnosis of graft rejection is made via liver biopsy and graded in severity via the Banff criteria (47, 48). In addition to criteria for typical TCMR and chronic rejection, the 2016 update of the Banff working group recognized what had previously been termed *de novo* autoimmune hepatitis as a form of plasma cell rich rejection and added criteria for the diagnosis of acute and chronic AMR (48). Modern immunosuppressive agents have resulted in a reduction of early acute rejection from 60 to 33.5% (49, 50). This finding concurs with other authors that reported TCMR to occur most commonly in the early post-transplant period (47). Early TCMR is a result of the direct alloantigen presentation pathway and is characterized by pleomorphic portal inflammation, bile duct injury and the lack of necro-inflammatory interface activity (48). The indirect alloantigen presentation pathway is thought to result in the late TCMR and has predominantly mononuclear portal inflammatory change, less subendothelial inflammation than early TCMR but more interface and necro-inflammatory perivenular activity (48, 51). Early TCMR generally responds to treatment and graft loss as a result is reported to be <1% (52). Late TCMR is less responsive and a preceding episode of moderate-severe early TCMR has been identified as a risk factor, however Jadloweic et al. reported more than half of the patients who experience late TCMR had no history of early TCMR (49). The implications of late TCMR are more sinister with a higher rate of graft loss due to chronic rejection or cholestasis (49). Chronic rejection occurs relatively rarely with a reported rate of 3–5% following liver transplant (53). Chronic rejection is defined as 50% bile duct loss and/or a foam cell arteriopathy, it typically occurs early following non-responsive acute rejection, and is increasingly recognized to have an antibody mediated component (48, 53).

Acute AMR causes graft dysfunction due to donor specific antibody (DSA) interaction to antigens on the graft. DSAs may be pre-existing (preformed) or develop post-transplant in response to foreign antigen (*de novo* antibodies). DSAs may be against HLA antigens, which are the most readily detected by current assays, or non-HLA antigens such as anti-glutathione S transferase (GSTT-1) and anti-angiotensine 2 receptor (54, 55). The development of anti GSTT-1 antibodies has been demonstrated to occur in recipients who are negative for the GSTT-1 gene but receive a graft from a GSTT-1 positive donor (55). These anti-GSTT1 antibodies have been shown to be pathogenic and are implicated in periportal inflammation, fibrosis and the loss of bile ducts (55). The understanding of AMR is evolving, it is also believed to often occur concurrently with ACR. The liver exhibits strong ABO and MHC I antigen expression on all liver cells in normal circumstances, however the MHC I expression on hepatocytes is weaker (56). Liver allografts in comparison to kidneys are highly resistant to HLA alloantibodies and numerous mechanisms are proposed to explain this phenomenon (57). Secretion of soluble HLA class 1 molecules which form immune complexes with alloantibodies and then subsequently undergo clearance by Kupffer cells is one such mechanism (57). Davies et al. demonstrated that the liver graft also continues to deliver HLA class I antigens into the recipients serum for the lifetime of the graft, thus generating called DSAs (58). Resistance to AMR is also enhanced by the fenestrated endothelium of the sinusoidal network as occlusion by activated immune complexes does not result in the same degree of ischaemia as other transplanted organs (23). The main clinical manifestations are graft dysfunction, transaminitis, and thrombocytopenia (48). The histological changes that occur are oedema, endothelial cell swelling, leukocyte sludging or margination and vascular deposition of tissue complement component 4d (C4d) (48). The catastrophic Vasculitis and intravascular thrombosis associated with hyperacute rejection of renal allografts is exceedingly rare following liver transplant (44, 59). Approximately 13% of liver transplant recipients have persisting DSA positivity and the most commonly found is the anti-HLA class II DSAs (60, 61). Del bello et al. found in a cohort type study that 5 of out the 21 subjects with *de novo* DSA formation experienced acute AMR and the average liver fibrosis score was higher in this subjects with DSAs (60). This latter finding is similar to previous authors who have associated DSA positivity with progressive fibrosis, graft loss and poorer patient survival (48, 61). Anastomotic biliary strictures have also been associated with the presence of anti-HLA class II DSAs in patients who have undergone ABO compatible transplantation (62). Rationale for this observation is that biliary structures receive their entire blood supply from the peri-biliary capillary plexus and therefore is not protected from occlusion by immune complexes in the same manner as the hepatic sinusoids (23). Establishing the histopathological evidence for the entity of chronic AMR is frequently challenging due to confounding factors (48). The Banff working group has established criteria for probable and possible chronic AMR. The histology findings associated with chronic AMR are low levels of portal, periportal, perivenular lymphoplasmacytic inflammation and interface necro-inflammatory activity with non-inflammatory fibrosis (48).

## The Immune Response to Cadaveric Grafts

During the initial decades of OLT, only cadaveric grafts from DBD donors were utilized. DCD programs emerged to expand the organ donor pool and consequently reduce waitlist mortality (63). At present, nearly a third of organ donations in the UK occur following DCD and the proportion of liver transplants utilizing DCD organs increased from 6.3% in 2005 to 26.3% in 2010 (64, 65). The inferior outcomes of DCD liver grafts was their higher rate of of PNF, non-anastomotic biliary complications, graft loss and poorer overall survival (65). However, the complication profile has changed over time with increased experience. Ischaemic injury occurs to the biliary epithelium during the dWIT making these grafts more susceptible to ischaemic cholangiopathy (65). It has been demonstrated that DCD grafts experience a more severe PRI with greater elevation of alanine transaminase (AST) and cell death (66). This may result in the devastating consequence of primary non-function (67). Despite DBD donation avoiding a period of circulatory arrest and subsequent warm ischaemia, significant detrimental changes are already thought to have occurred within the graft as a result of brain death. The physiological changes that take place during brain death have been described as an “autonomic storm” with initial intense parasympathetic response followed by short lived sympathetic activation (68). The decline in sympathetic activity is accompanied by myocardial depression and at all stages of this process the liver is subjected to an ischaemic type injury (68). In addition, there is widespread activation of inflammatory mediators irrespective of any hemodynamic instability (69).

An experimental animal study utilizing a rat model investigated the differing proinflammatory (TLR4, HMGB1, IL-1β, IL-6, TNF-α, MCP-1, E-selectin, and P selectin) cytoprotective (HO-1, VEGF, Hif-1α) and injury gene (P21, Bax, Bcl-2) expression associated with DCD and DBD grafts, utilizing a living donor liver as a reference (70). Directly after organ retrieval, DBD grafts demonstrated a down regulation of TLR4 but an upregulation of IL-6 (326-fold), IL-1β (15-fold), TNF-α (22- fold), P-selectin (41.7-fold), and E-selectin (12.9- fold) in comparison to LD grafts (70). The DCD livers only demonstrated an increase in HMGB1 directly after retrieval, in comparison to the living donor group. In addition, HO-1 expression increased to a larger extent in the DBD (12-fold) than the DCD (5.6-fold) livers in comparison to the living donor liver. As indicated by gene expression, the DBD and DCD grafts responded differently a period of cold ischaemia. After 12 h of cold ischaemia, the DBD livers inflammatory gene expression did not change significantly from immediately post retrieval. However, DCD Livers demonstrated a 4-fold increase in IL-6, 30-fold increase in MCP-1 and 4-fold increase in E-selectin in comparison to living donor grafts (70). The pro-apoptotic gene Bcl-2 increased significantly (4.6-fold) in the DCD livers in comparison to both DBD and liver donor livers. DBD livers showed a further 17-fold increase in HO-1 gene expression after a period of cold ischaemia in comparison to the living donor grafts. These findings demonstrate a pronounced inflammatory process is occurring in the liver at the time of retrieval in DBD livers, likely as a result of the physiological and inflammatory changes that occur during brain death. It was proposed that not enough time had elapsed following the short but significant period of warm ischaemia in the DCD livers to see a significant increase in inflammatory and apoptotic genes at the time of retrieval.

A recent cohort study that compared DCD and DBD grafts demonstrated equivalent outcomes in regards to primary non-function, acute cellular rejection, need for retransplantation and patient survival at 3 years (71). Pitarch Martinez et al. (71) demonstrated an acute cellular rejection rate of 20% in DCD and 16.4% in DBD grafts that was not statistically significant (*P* = 0.685) (71). However, in this cohort the DCD grafts needed to meet strict criteria (Maastricht III, WiT <30 min, Donor age ≤65) and their recipients had lower MELD scores. Doyle et al. performed a similar cohort type study and had similar findings with the rate of rejection being 24.5 and 26.5% in the DCD and DBD group, respectively (*P* = 0.84), the early rejection rate (≤30 days) was identical (72). A case matched study by Pine et al. comparing DBD to DCD grafts also demonstrated a similar rate of both acute and chronic rejection however primary non-function was higher in the DCD group (2/39 vs. 0/39) (73). PRI is thought to be increased in DCD grafts and this has been demonstrated by a greater elevation in early post-operative transaminases (74). Despite innate immunity being the main driver of PRI, it has been suggested that this is positively correlated with subsequent graft rejection (75). Mechanisms for this include trafficking of DCs into the graft and enhanced T cell priming (37). The results of the aforementioned clinical studies have not demonstrated this effect.

A study by Xystrakis et al. investigated the frequency and function of T-cell subsets in liver perfusate fluid obtained from DBD, DCD and living donors. The perfusate fluid was obtained from the graft at the end of the cold storage period and was analyzed by flow cytometry, cell sorting and culture (76). The frequency of memory and naïve T-cell subsets in the perfusate did not differ between all graft types but the frequency of CD69^+^ CD8 T-cells was significantly higher in the perfusate from DBD grafts (76). In addition, the proportion of IL-2 and IFN-γ produced by CD8 T-cells was higher in DBD grafts. These authors concluded that the process of brain death is associated with the release of non-specific inflammatory mediators (76). Jaseem et al. compared immunohistochemical findings of preimplantation liver biopsies from living and deceased (DBD) donor grafts (77). Significantly higher levels of CD3^+^ lymphocytes and Kuppfer cells were found in the DBD grafts. In addition, the adhesion molecule ICAM-1 was found to be expressed at higher levels in the DBD grafts (77). A higher percentage of CD3^+^ lymphocytes in the preimplantation biopsy was associated with subsequent acute rejection in the DBD graft recipients (77). These authors concluded that the process of brain death resulted in a significant increase in inflammatory cell recruitment and migration into the liver allograft in comparison to living donor grafts. However, clinical outcomes of the recipients did not differ (77).

The literature describing humoral responses in ABO compatible DCD grafts is sparse. Levitsky et al. (78) compared the differences of both preformed and *de-novo* DSAs in living donor with deceased donor recipients, however the results for DCD and DBD subgroups were not published (78). This study did not demonstrate a difference in either preformed or *de-novo* DSA formation in either graft group. The presence of DSAs, either preformed or *de-novo*, did not affect patient survival in either graft group but did affect the graft survival (78). The deceased donor recipients with *de-novo* DSAs had higher rates of graft failure (*P* = 0.005) (56). Coexisting TCMR or recurrent viral hepatitis is thought to increase the DSA mediated damage as inflammation within the liver increases MHC I expression and induces MHC II expression. As previously mentioned, DSAs can be directed at either of these MHC molecules. Inlet and mononuclear septal venulitis have been suggested as the cause of the interface hepatitis that occurs with the presence of *de novo* DSA formation (56).

## The Immune Response to Living Donor Grafts

The lack of suitable deceased donor livers for transplantation and the associated waitlist mortality has prompted the development of living donor liver transplantation (LDLT). The first successful LDLT was performed in Australia in 1989, a female adult donated her left lobe and it was implanted into her to 17-month-old son who suffered from biliary atresia (79). Following this pivotal event, LDLT has been performed around the world and at present one third of pediatric liver transplants involve a living donor (80). Particular political, cultural and religious beliefs in Asian countries have resulted in very low rates of deceased donors but the highest rates of LDLT (81, 82). Initially, LDLT procedures were limited to adult-pediatric with left lateral segment grafts (81). Significant progress has occurred and at present adult-adult LDLT with right lobe grafts are now being performed (81). LDLT is technically challenging as the graft must have an adequate volume of parenchyma, portal and arterial inflow, venous outflow and biliary drainage. The transplantation of a substantially smaller hepatic allograft in LDLT puts the recipient at the additional risk of small-for-size-syndrome (SFSS) (82). Other additional risks inherent with LDLT are the surgical risks posed to the donor (83). Inference based on the experience from living donor kidney transplantation would suggest that LDLT would have superior immunological outcomes, however this is yet to be conclusively demonstrated (84).

In the United states, adult-to-adult LDLT is increasing in frequency with a 82% graft survival at 1 year and a 10 years overall survival post-transplant that exceeded deceased donor transplantation (70 vs. 64%) (83). Avoidance of a graft exposed to the physiological perturbations of brain death and minimal cold ischaemic time are both thought to reduce the initial inflammatory response and subsequent immune activation. In addition, there may be HLA matching between genetically related donors and recipients (84). The evidence regarding the immunological benefits of adult-to adult LDLT is conflicting at present. Shaked et al. demonstrated in their retrospective review a similar rate of biopsy proven acute cellular rejection, more recurrent episodes and more frequent graft loss as a result in LDLT in comparison to deceased donor transplants (84). Subsequent to this, Levitsky et al. demonstrated that the incidence of acute cellular rejection was significantly lower in LDLT patients who received a graft from a biologically related donor in comparison to a non-biologically related and deceased donors (85). Another pertinent finding from this study relating to all liver transplant recipients was that an episode of biopsy proven cellular rejection significantly increased the patients risk of subsequent graft loss and death. The humoral immune response following LDLT has also been investigated by Levitsky et al. (78) and these authors found no difference in preformed or *de novo* donor specific antibody formation in LDLT in comparison to cadaveric graft recipients (DSA) (78). It was demonstrated however that *de novo* DSA positivity was associated with higher graft failure in both LDLT and cadaveric graft recipients and this relationship was proportional to the quantity of DSA present.

Experimental data indicates that inflammatory cell and cytokine concentrations are significantly lower in living donor grafts prior to retrieval, reperfusion and post reperfusion in comparison to brain death donors (68). Liver biopsies taken at various timepoints during the retrieval and transplant procedure by Weiss et al. (68) demonstrated that the mRNA concentration of CD3 and CD25 to be significantly lower in LDLT grafts in comparison to those from DBD donors. These authors concluded that although the presence of immune cells and cytokines increase as the LDLT procedure progresses, the level of immune activation is far less intense than that in transplants with DBD grafts (68). Interestingly, the LDLT recipients in this study had significantly lower transaminases in the post-operative period compared with DBD recipients which signifies a milder PRI. These authors also demonstrated a lower incidence of biopsy proven rejection in the 24 months post-transplant for LDLT in comparison to DBD graft recipients (38 vs. 28%, *P* = *0.04*) (68). De Jonge et al. performed biopsies on both cadaveric and living donor grafts prior to retrieval (PRE), following cold perfusion (COLD) and post reperfusion in the recipient (POST) (86). Gene expression was analyzed and there was an upregulation of inflammatory genes between the PRE and POST biopsy in the cadaveric grafts, these included genes for IL-8 and ICAM-1. In the living donor grafts there was also an upregulation of genes for SOCS3, Hepatocyte growth factor (HGF) and NFκB1 from the PRE to the POST reperfusion sample and these are all associated with regeneration (86). The parenchymal transection during living donor procurement may be the initiating stimulus for this. There was also upregulation of MHC II genes in the living donor grafts and it was suggested that smaller grafts are associated with increased alloreactivity (86).

## ABO Incompatible Grafts

Transplanting organs across the ABO blood groups has for a long time been associated with poor outcomes due to increased graft loss and worse patient survival (87). The blood antigens are expressed on hepatic vasculature, biliary epithelium and hepatocytes and all are a target for AMR. Despite knowledge of these reactions and the inferior results, a compatible graft may not be available and an emergency situation may necessitate ABO incompatible transplant to prevent certain death (88). Since its initial inception, numerous immune modulating strategies and therapies have been implemented and a recent meta-analyses found no difference in patient survival following an ABO incompatible in comparison to an ABO compatible transplant (89, 90).

Acute AMR is a feared consequence of ABO-I liver transplantation and can often lead to loss of the graft. Numerous interventions have been attempted to mitigate the risk of AMR and these include; preoperative plasmapheresis, splenectomy, local infusions, mycophenolate mofetil, and rituximab (91) Several studies have failed to demonstrate a correlation between preoperative ABO antibody titer and AMR (92). It has been the implementation of rituximab, an anti-CD20 monoclonal antibody that has yielded the greatest improvement in outcomes (92–94). Commonly used AMR prophylaxis regimes include a single dose of Rituximab 2–3 weeks prior to transplantation (92, 94). Plasmapheresis aims to reduce the ABO antibody titer and is commonly performed both prior to transplantation and post operatively, however the antibody titer level targeted with this modality varies between institution (92). Undertaking a splenectomy on the recipient at the time of transplantation initially gained acceptance as this organ is the site of antibody production and harbors a large amount of B cells and plasma cells (92). However, studies have failed to demonstrate a benefit from this procedure, especially following the introduction of rituximab (92, 93).

Acute AMR in the ABO incompatible graft can result in graft failure via two types of injuries; liver necrosis in the first 1–2 post-operative weeks or diffuse intra-hepatic bile duct injury in the subsequent 2–3 months (92). These injuries are thought to occur because the hepatic vascular endothelium and biliary epithelium exhibit ABO antigens and therefore are sites for antibody-antigen binding with subsequent complement activation, cytokine production, cell migration and thrombus formation (93). The biliary damage manifests as diffuse biliary strictures and can result directly from the antibody-antigen reaction or secondary to ischaemia from intrahepatic arterial thrombosis (ischaemic cholangiopathy) (94). Song et al. compared outcomes in a cohort of patients that underwent either ABO compatible LDLT or ABO incompatible LDLT following the introduction of Rituximab in the desensitization protocol (94). The difference in patient survival, biopsy proven TCMR and post-operative LFTs were not significant. Biliary strictures were more common in the ABO incompatible group (20.7 vs. 14.2%, *p* = *0.038*) with non-anastomotic biliary strictures occurring in 12 (8.5%) of the ABO incompatible recipients. Interestingly, all 12 underwent liver biopsy and only one case had histopathological evidence of AMR. No recipient of an ABO compatible graft developed a non-anastomotic biliary stricture (94).

## Immune Response in the Pediatric Patient

The outcomes of pediatric liver transplantation have improved significantly over the last several decades and a 2012 study demonstrated a 1 and 10 years survival of 95 and 88%, respectively (95, 96). The indications for transplantation differs in the pediatric population with the most common indication in the US being biliary atresia (95, 97). In contrast to adults, transplantation for viral hepatitis and malignant tumors are a rare occurrence. Approximately 8% of liver transplants each year in the US are performed on children with an equal portion receiving either a whole or split graft (98). A large Canadian transplant center reported LDLT to comprise 46% of all pediatric LT. The same authors reported that the LDLT grafts comprised the left lateral section, left and right lobe in 82.8%, 3.7% and 13.4% respectively between 2000 and 2015 (99). Operational tolerance, post-transplant lymphoproliferative disorder (PTLD) and graft fibrosis occur more commonly in this patient population.

An individual's immune system needs to rapidly adapt as it makes the transition from intra-uterine life to the antigen rich outside world (100). Initially there is a heavy reliance on innate mechanisms, with maturation of the immune system gradually occurring as we progress through childhood. Acute cellular rejection is a common occurrence in this population with reported incidence of up to 60% (95). In a retrospective cohort study of 46 children who received a split graft from a living relative, 44 episodes of ACR occurred over 10 years of follow up with 35 of these in the first 6 weeks post op (101). It is believed that younger children (<1 year of age) are more likely to become immunologically tolerant of their graft, but the mechanism is yet defined (102, 103). Byun et al. compared outcomes of pediatric recipients that underwent OLT at <12 months of age with those older than 12 months and found the rate of ACR to be similar (30.2 and 33.0%, *P* = 0.848) in each group (104). This differs from a previous study utilizing the SPLIT database which demonstrated the rate of ACR in those <12 months was significantly less than in older pediatric recipients (0.20 vs. 0.44 episodes per patient-year, *P* = 0.001) (105). A large retrospective cohort study by Talisetti et al. assessed numerous factors and their relationship with operational tolerance (106). A recipient age of <12 months was the only variable significantly associated with a higher rate of developing operational tolerance (106). It has been demonstrated that in early infancy the Th2 cytokines (IL4, IL10) predominate over the Th1 cytokines (IL-2, IFN-γ) and this may contribute to graft acceptance in this age group (107). This is supported by the fact that pediatric patients that experienced TCMR had a higher proportion of Th1 cytokines (107).

Post-operative frequency of TREGs and IL-4 are higher in pediatric recipients who receive a LD graft in comparison to a deceased donor graft cadaveric (108). Favorable immunological outcomes would be expected as TREGs and IL-4 are both associated with immunotolerance, however the clinical evidence is less clear. In the retrospective cohort study published be Kehar et al. compared TCMR rates between pediatric LD and deceased donor recipients. The 1, 3 and 5 years TCMR rejection free survival was 64.4%, 61.1%, and 61.1% for LD and 55%, 44.4%, and 43.4% for cadaveric graft recipients respectively, however this difference was not statistically significant (*P* = 0.08*)* (99). Alonso et al. found that the incidence of rejection was the same in pediatric cadaveric graft compared to LD recipients (78 vs. 74%) but there was a higher rate of steroid resistant rejection in the cadaveric graft group (43 vs. 13%, *P* ≤ 0.01). Kehar et al. found no difference in rejection rates between pediatric patients that received a graft from a genetically related compared with unrelated donor (*P* = 0.4) (99).

## Implications for Clinical Practice

There is variation in both the standard post-operative immunosuppression and rejection treatment regimens utilized by transplant centers around the world. The immunosuppressant drug most commonly used long term is the calcineurin inhibitor, tacrolimus, based on evidence of improved efficacy (109, 110). There does not appear to be clinical evidence that altering standard immunosuppressive regimes based on the type of graft is beneficial, the only exception to this would be the implementation of pre-operative Rituximab infusions for ABO-I transplants. Pediatric patients that are transplanted at <12 months of age appear to have a more immunotolerant profile. This should be considered when planning transplantation for their native liver disease as it may optimize graft survival and minimize morbidity from immunosuppression. The inflammatory insult on the hepatic allograft that occurs during the period of brain death is undeniable. Administration of corticosteroids to the donor prior to organ retrieval has been investigated in a randomized controlled trial but provided no benefit (111). Further research is needed in this area as it may improve outcomes and result in the increased utilization of grafts. The desire to minimize graft injury during the preservation period has led to the development of machine perfusion strategies such as hypothermic (HMP) and normothermic machine perfusion (NMP). A recent randomized controlled trial of NMP demonstrated increased organ utilization and a reduction in preservation-reperfusion injury, as evidenced by a significant reduction in post-operative LFTs (112). Administration of anti-inflammatory and immune mediating therapies directly to the hepatic allograft via the machine perfusion circuit is a growing area of research interest (113).

Tolerance inducing therapies are showing promising results in clinical studies. In a landmark pilot study, Todo et al. showed that a single post-operative infusion of TREG cells allowed accelerated withdrawal of immunosuppression at 6 months post LDLT and 70% of these individuals achieved operational tolerance (114). The patients that experienced rejection in this pilot study were transplanted for AILD, suggesting that these individuals may require additional strategies (114). This study utilized recipient TREG cells that were co-cultured with irradiated donor leukocytes obtained several weeks before transplantation, an opportunity that does not exist in the deceased donor transplantation setting. The participants also underwent splenectomy at the time of transplantation. Sanchez-Fueyo et al. recently published results of a phase 1 clinical trial investigating the safety, applicability and biological activity of treg administration post cadveric liver transplantation (115). The treg cells in this study are autologous and not exposed to donor antigens during the culture process Subjects in this study received a doses of either 0.5–1 or 3–4.5 million tregs/kg. Nine subjects were enrolled and only a single subject who received the higher dose experienced a transfusion reaction (115). The frequency of tregs in the peripheral blood of subjects who received the higher dose remained elevated for 1 month and this likely reflected the infused tregs as the subpopulation that increased was similar to the infused cells (115). Although it did not reach statistical significance, donor specific hyporesponsiveness in the group that received the larger dose of tregs was observed (115). Tregs exert their suppressive effects on multiple different immune cells via both direct and indirect mechanisms (116). Direct mechanisms include IL-10, IL-35, TGF-β, secretion which results in apoptotic cell death of target effector cells. Depletion of extracellular ATP and IL-2 via the expression of CD39/CD73 and CD25, respectively, are examples of the indirect mechanisms (117). Other tolerance inducing therapies currently under investigation include Dendritic Cells, IL-2 and regulatory macrophages. Their rationale and current place in clinical transplantation are outlined elsewhere (118, 119).

## Conclusion

The field of liver transplantation has advanced significantly since the 1960's. Progress has been made in organ preservation, post-operative care, immunosuppression and optimal utility of different grafts to ensure those in need get the best possible access to this lifesaving procedure. Grafts previously not possible such as ABOi LDLT are now commonplace in many centers with acceptable results. Based on existing literature, a similar immune response is elicited to the majority of grafts following implantation, comprising an initial innate inflammatory response due to preservation-reperfusion mechanisms followed by a predominantly cell mediated response. However, contribution from the innate-like and humoral components of the immune system to PRI and graft rejection are becoming increasingly recognized. Grafts from brain dead donors appear to have a higher inflammatory cell infiltrate and cytokine concentration at retrieval than DCD or living donor grafts, however clinical differences in rejection as a result are not evident. It is likely that both cell and antibody mediated injury results in the morbidity associated with chronic rejection. Acute TCMR is common following OLT but undergoing transplant in the first year of life seems protective. Hyperacute graft rejection of the liver is exceedingly rare. Strong clinical evidence that a DBD, DCD or LDLT graft is associated with a lower rate of TCMR is not apparent. Research into tolerance inducing therapies has shown promising results and the results of larger, phase II trial are eagerly awaited.

## Author's Note

AH was employed by the University Hospital Birmingham NHS Trust as a clinical research fellow.

## Author Contributions

AH and D-CO-B performed the literature search and constructed the manuscript. DN, VR, SW, and MP reviewed and contributed to the manuscript. All authors contributed to the article and approved the submitted version.

## Conflict of Interest

The authors declare that the research was conducted in the absence of any commercial or financial relationships that could be construed as a potential conflict of interest.
